# Optimizing Running Mechanics, Effects of Cadence, Footwear, and Orthoses on Force Distribution: A Quasi-Experimental Study

**DOI:** 10.3390/jfmk10010089

**Published:** 2025-03-10

**Authors:** Marie Adelaide Nicolas-Peyrot, Yves Lescure, Eleonore Perrin, Magdalena Martinez-Rico, Corentin Travouillon, Gabriel Gijon-Nogueron, Eva Lopezosa-Reca

**Affiliations:** 1 Facultad Ciencias de la Salud, Universidad de Malaga, 29017 Malaga, Spain; ma-nicolaspeyrot@ecole-rockefeller.com (M.A.N.-P.); yves-lescure@ecole-rockefeller.com (Y.L.); 2 Department of Podologie, Ecole Rockefeller, 69008 Lyon, France; eleonore-perrin@ecole-rockefeller.com; 3 Department of Health Sciences, Faculty of Nursing and Podiatry, Industrial Campus of Ferrol, Universidad de da Coruña, 15001 Ferrol, Spain; magdalenamr96@gmail.com; 4 TRINOMA Co., 48800 Villefort, France; corentin.travouillon@gmail.com; 5 Department Nursing and Podiatry, Universidad de Malaga, 29017 Malaga, Spain; evalopezosa@uma.es

**Keywords:** running, biomechanics, cadence, foot orthoses, plantar pressure

## Abstract

**Background**: Running is a popular physical activity known for its health benefits but also for a high incidence of lower-limb injuries. This study examined the effects of three biomechanical interventions—cadence adjustments, footwear modifications, and foot orthoses—on plantar pressure distribution and spatiotemporal running parameters. **Methods**: A quasi-experimental, repeated-measures design was conducted with 23 healthy recreational runners (mean age 25, mean BMI 22.5) who ran at least twice per week. Five conditions were tested: baseline (C0), increased cadence (C1), orthoses (C2), low-drop footwear (C3), and a combination of these (C4). Data were collected on a Zebris treadmill, focusing on rearfoot contact time, peak forces, and stride length. **Results**: Increasing cadence (C1) reduced rearfoot impact forces (−81.36 N) and led to a shorter stride (−17 cm). Low-drop footwear (C3) decreased rearfoot contact time (−1.89 ms) and peak force (−72.13 N), while shifting pressure toward the midfoot. Orthoses (C2) effectively redistributed plantar pressures reducing rearfoot peak force (−41.31 N) without changing stride length. The combined intervention (C4) yielded the most pronounced reductions in peak forces across the rearfoot (−183.18 N) and forefoot (−139.09 N) and increased midfoot contact time (+5.07 ms). **Conclusions**: Increasing cadence and low-drop footwear significantly reduced impact forces, improving running efficiency. Orthoses effectively redistributed plantar pressures, supporting individualized injury prevention strategies. These findings suggest that combining cadence adjustments, footwear modifications, and orthoses could enhance injury prevention and running efficiency for recreational runners.

## 1. Introduction

Running holds an essential place in the sporting landscape and continues to attract an increasing number of participants worldwide, with more than 600 million people engaging in this activity. In recent years, its popularity has surged, particularly due to its accessibility and widely recognized health benefits. A 2022 report by World Athletics highlighted a 30% increase in running participation over the past decade, driven by urban running events and the rise of fitness culture [1,2]. In France alone, 25% of the population (12.4 million people) participate in running, underscoring its widespread appeal [3].

Beyond its popularity, running is associated with both physical and mental health benefits, including improved cardiovascular health, weight management, and stress reduction [4]. However, it also presents a notable risk of musculoskeletal injuries, with an annual incidence ranging from 19.4% to 79.3% among runners [5]. The most common injuries affect the lower limbs [6,7], particularly the knee, leg, and foot, resulting from multifactorial causes, including training errors, biomechanical characteristics, and environmental factors.

The most prevalent injuries include patellofemoral pain syndrome (PFPS), iliotibial band syndrome (ITBS), Achilles tendinopathy, and stress fractures. These injuries not only hinder performance but may also lead to long-term health consequences. Studies indicate that up to 50% of runners experience recurrent injuries within a year of returning to activity [8]. For instance, PFPS is often linked to patellar misalignment and muscle imbalances [9,10], ITBS is associated with excessive friction between the iliotibial band and femur [11,12], Achilles tendinopathy results from repetitive strain, and stress fractures are common in runners who dramatically increase their training volume [13,14].

These injury risks highlight the need for targeted biomechanical interventions that address improper force distribution and suboptimal spatiotemporal parameters during running [15,16]. Risk factors such as inappropriate training loads and biomechanical abnormalities (e.g., overpronation, excessive hip adduction) have been implicated, though their direct relationship with injuries remains debated [17,18,19,20].

Recent research emphasizes the role of running technique, particularly cadence and foot strike patterns, in modulating injury risk. A low cadence or pronounced rearfoot strike has been associated with increased knee and hip loading, contributing to PFPS and ITBS [21,22]. Consequently, running retraining has emerged as a promising strategy for reducing injury risk by optimizing biomechanical parameters and redistributing forces away from vulnerable structures [23,24].

While previous studies have examined cadence modification, footwear adjustments, and orthoses separately, little is known about their combined effects on running mechanics. Understanding these interactions could improve injury prevention and rehabilitation strategies. While studies have examined their separate effects, limited evidence exists on their synergistic impact [25,26].

This study aims to assess both the individual and combined effects of these interventions on key biomechanical parameters, such as cadence, stride length, and force distribution. By addressing this gap in the literature, this research seeks to provide evidence-based recommendations for optimizing running mechanics, reducing injury risk, and enhancing performance.

Biomechanical running analysis, including detailed stride assessment and correction of abnormalities, has shown promise in injury prevention. Advanced tools, such as the Zebris instrumented treadmill, allow for real-time plantar pressure analysis, identifying spatiotemporal gait parameters and force distribution patterns. These include cadence, stride length, rearfoot–midfoot–forefoot contact time, and peak force distribution, essential for designing targeted interventions to correct biomechanical inefficiencies [27].

Several biomechanical interventions have been proposed to mitigate injury risk, including cadence modifications, footwear adjustments, and foot orthoses [28,29]. Increasing cadence (typically by 5–10%) has been shown to reduce stride length, vertical oscillations, and braking forces during ground contact [30,31]. Similarly, low-drop footwear promotes midfoot or forefoot strikes, shifting mechanical loads away from the knee [32,33]. Foot orthoses modify plantar pressure distribution and foot structure deformation, but their precise effects remain uncertain—especially when combined with other interventions [34,35].

This study seeks to provide a comprehensive understanding of these biomechanical interventions and their potential for reducing injury risk and enhancing running performance. The findings will offer valuable insights for clinicians and sports scientists in optimizing running mechanics and promoting long-term athlete health.

## 2. Methods

### 2.1. Protocol and Registration

This study complied with all STROBE guidelines [29]. This study was conducted in accordance with the Declaration of Helsinki on Ethical Principles for Medical Research Involving Human Subjects and was approved by the Ethics Committee of the University of Malaga (CEUMA 206-2023-H) in Spain [30]. Data confidentiality was also ensured. Data collection and storage adhered to strict confidentiality protocols. To ensure data confidentiality, all personal identifiers were removed or anonymized. Participant data were securely stored in an encrypted database, accessible only to the research team. No data will be publicly shared but may be made available upon reasonable request, in compliance with legal and ethical regulations.

### 2.2. Design

A quasi-experimental design with repeated measures was chosen for this study. This approach allowed for the evaluation of multiple interventions within the same group of participants, thereby reducing inter-individual variability and increasing statistical power. While randomized controlled trials (RCTs) are often considered the gold standard for reducing bias, a quasi-experimental design was deemed more practical due to logistical constraints, such as the difficulty of recruiting a large number of participants for multiple randomized groups.

Additionally, this design enabled the assessment of combined interventions (e.g., cadence adjustment, footwear modification, and orthotics), which would be challenging to implement in a traditional RCT framework. To address potential sources of bias, strict control over experimental conditions was maintained, including standardized warm-up protocols, rest periods, and environmental factors.

Each participant was randomly assessed under the following five conditions (Table 1):

- C0 (Reference Condition): Rearfoot-to-toe drop of 10 mm, no cadence adjustment, no orthotics.
- C1 (Cadence only): 10 mm rearfoot-to-toe drop, 10% increase in cadence, no orthotics.
- C2 (Plantar orthoses): 10 mm rearfoot-to-toe drop and Alain Lavigne Inversion Foot Orthoses (ALIFOrthoses) [31].
- C3 (Asics Noosa shoe): 5 mm rearfoot-to-toe drop, no orthotics. The low drop favors a plantar attack on the forefoot or midfoot, which can modify the forces exerted on the rearfoot compared to high-drop shoes [32].
- C4 (Cross-interventions): 10% increase in cadence, ALIFOrthoses, 5-mm rearfoot-to-toe drop.

### 2.3. Participants

A total of 23 healthy recreational runners (mean age: 25 ± 4.5 years, BMI: 22.5 ± 1.34) were recruited. Participants were required to run at least twice weekly and have no history of lower-limb injuries in the past six months. All participants were at least 18 years old and could follow the study instructions.

Inclusion criteria:

- Age ≥ 18 years.
- Recreational running activity of at least 2 sessions per week (running 5 km in under 25 min).
- Habitual use of running shoes with a 10 mm drop.
- Willingness and ability to adhere to the study protocol.

Exclusion criteria:

- Degenerative diseases of the bones and joints (diagnosed based on medical history).
- Surgery of the lower limbs.
- Recent knee or ankle injuries or serious foot injuries that may have left morphological changes.
- Painful skin conditions (e.g., calluses, plantar warts).
- Lower-limb injuries in the past 6 months (verified by self-report).

These criteria ensured the selection of a homogeneous population, minimizing potential biases linked to biomechanical disorders or adaptations.

After receiving detailed information about the study aims and procedures, each participant signed an informed consent form.

The experimental measurements were carried out at the R&D laboratory of the Rockefeller School in Lyon (8th arrondissement) between February and November 2024. The laboratory was equipped to maintain controlled environmental conditions, optimizing data reliability.

### 2.4. Procedure

**Figure 1:**
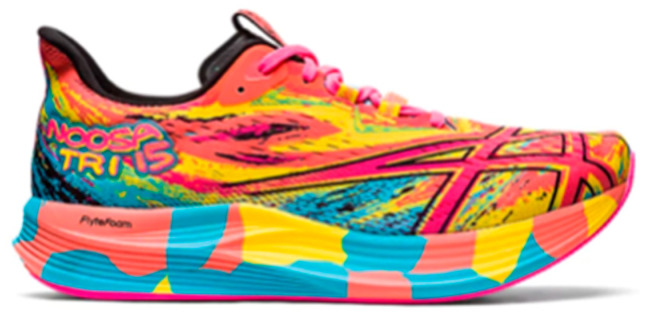
Profile view of the Noosa model from Asics.

The running shoes used in the study were ASICS NOOSA, featuring a 5 mm rearfoot-to-toe drop and a weight of 255 g (men’s size 9). They incorporated FlyteFoam^®^ (Onitsuka Co., Ltd., Kobe, Japan) midsole technology for lightweight cushioning with organic fibers to enhance durability. The outsole was made of wet grip rubber and was designed to provide excellent traction on wet surfaces, while the upper part comprised a no-sew, breathable mesh for superior comfort (Figure 1).

**Figure 2:**
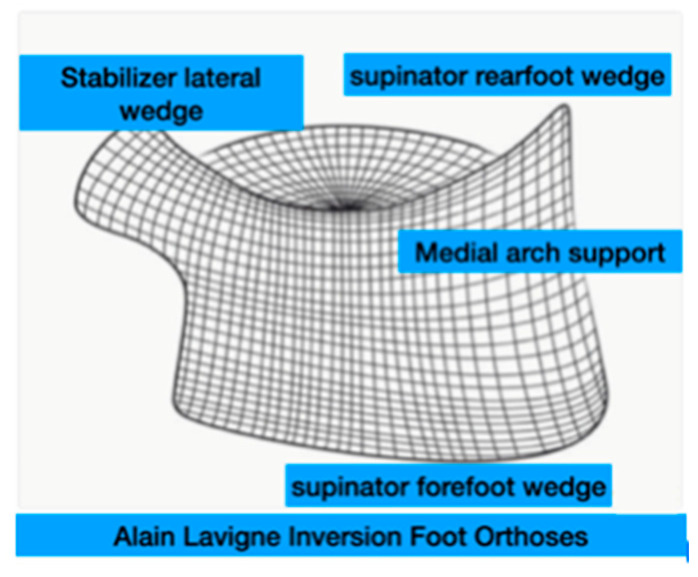
Front view of Alain Lavigne Inversion Foot Orthoses design.

The foot orthoses were thermoformed “Alain Lavigne Inversion Foot Orthoses” (ALIFOrthoses) [31], designed with a full-length medial wedge and a Shore A hardness of 35. The orthoses included a supinating rearfoot wedge, medial arch support, supinated forefoot wedge, and a lateral stabilizing wedge for enhanced support and alignment (Figure 2).

### 2.5. Experimental Conditions

The Zebris treadmill was selected for its unique ability to measure plantar pressure distribution and spatiotemporal parameters in real time. The key features include a matrix of capacitive pressure sensors under the tread, which provides high-resolution data on force distribution and gait dynamics. This system allows precise speed adjustments (0.2–24 km/h in 0.1 km/h increments) and real-time data visualization, making it ideal for analyzing running mechanics under controlled conditions.

Before starting the experimental conditions, the participants completed a 5 min warm-up period on the Zebris treadmill at a self-selected speed while wearing their own shoes. This was performed to ensure participant comfort and achieve a stable running state, thereby reducing potential variability during the trials, in line with the recommendations of similar studies.

To minimize fatigue as a confounding factor, rest periods of five minutes were implemented between conditions. During this time, participants remained seated, hydrated, and refrained from physical activity.

Each participant ran at a fixed speed of 12 km/h on a Zebris treadmill, which was selected for its high-resolution plantar pressure measurement and spatiotemporal parameter analysis.

Each participant performed test runs under the five distinct experimental conditions described above, each performed at a constant speed of 12 km/h. This speed was chosen based on its relevance to recreational runners because it represents a moderate pace that is achievable for most participants while allowing for detailed biomechanical analysis. However, this fixed speed may limit the external validity of the findings for runners who typically train at significantly faster or slower paces.

Biomechanical data from the Zebris treadmill is collected using a matrix of capacitive pressure sensors integrated under the tread. These sensors continuously measure the pressure exerted by the feet on the tread surface. They are individually calibrated to ensure high accuracy [33,34]. Data were acquired via sensors that recorded the variations in pressure as the subject ran on the treadmill. The data include information on the distribution of plantar pressure, phases of contact (rearfoot, midfoot, and toes), and zones of maximum pressure [34].

The measurements were transmitted in real time to a computer to ensure synchronization with the software. This dedicated software interprets and displays data in real time in the form of color-coded plantar pressure maps and spatiotemporal parameters, such as cadence, step length, contact time, etc., to analyze balance and stability [35].

The data are then recorded for in-depth analysis. The software generates graphs, tables, and detailed reports. The results are stored and can be compared across multiple trials or subjects to assess progress or detect asymmetries [33].

Each condition consisted of two minutes of running: the first minute was for familiarization and the second minute for data collection.

### 2.6. Sample Size

The required sample size was calculated using a 95% confidence level and 80% power based on the effect size derived from preliminary data. The study concluded that 24 participants per group were required to achieve the study objectives. However, due to recruitment challenges, the final sample size was limited to 23 participants. This discrepancy may slightly reduce the statistical power of the study, potentially increasing the risk of Type II errors (false negatives). Nevertheless, the repeated-measures design mitigates this limitation by increasing the sensitivity to detect within-subject differences.

### 2.7. Statistical Analysis

A repeated-measures ANOVA was chosen because each participant underwent all five conditions, allowing us to account for within-subject variability and enhance statistical power by using each individual as their own control. Before analysis, we checked normality to verify the normality of data distribution (*p* > 0.01). In cases where normality assumptions were violated, non-parametric tests (e.g., Wilcoxon signed-rank test) were applied to assess the effects across conditions while controlling for inter-individual variability. The Kolmogorov–Smirnov test was conducted to verify the normality of data distribution (*p* > 0.01). In cases where normality assumptions were violated, non-parametric tests (e.g., Wilcoxon signed-rank test) were applied.

Descriptive statistics were expressed as means and standard deviations, with 95% confidence intervals. Statistical significance was set at *p* < 0.05, and all analyses were performed using MATLAB (R2021a, 8.3.0.532, The MathWorks Inc., Natick, MA, USA), which was chosen for its robust handling of complex biomechanical data and capability to run post hoc comparisons with appropriate corrections for multiple testing.

## 3. Results

The study included 23 recreational runners with an average age of 25 years (SD = 4.5) and a BMI of 22.5 (SD = 1.34). Participants ran at least twice per week and had no history of lower-limb surgery or injuries in the past six months. To facilitate results interpretation, key findings are summarized before each table.

### 3.1. Overview of Key Findings

- Cadence adjustment (C1) and the combined intervention (C4) showed the most pronounced reductions in rearfoot peak force, while footwear changes primarily influenced midfoot contact time.
- The combined intervention (C4) produced the largest reduction in rearfoot impact (−139.09 N, *p* < 0.001), suggesting it may be the most effective strategy for minimizing joint stress in runners.
- Footwear modification (C3) led to an increase in midfoot contact time (+1.98 ms), while orthoses (C2) redistributed plantar pressure with a moderate effect on rearfoot force.
- Stride length and step length were significantly reduced under conditions C1 and C4, suggesting a more compact and controlled gait pattern.

### 3.2. Spatiotemporal and Force Distribution Comparisons

A detailed comparison of spatiotemporal parameters and force distribution across conditions is presented in Table 2.

### 3.3. Key Observations

Regarding rearfoot contact time, condition C2 reduced it by 1.22 ms (Cohen’s d = 0.45, *p* < 0.001), indicating a moderate effect. In contrast, condition C3 increased rearfoot contact time by 1.89 ms (Cohen’s d = 0.61, *p* < 0.001), reflecting a more noticeable change. With respect to the midfoot contact time, C4 had the most substantial impact, increasing this phase by 5.07 ms (Cohen’s d = 0.89, *p* < 0.001), suggesting a strong effect.

**Figure 3:**
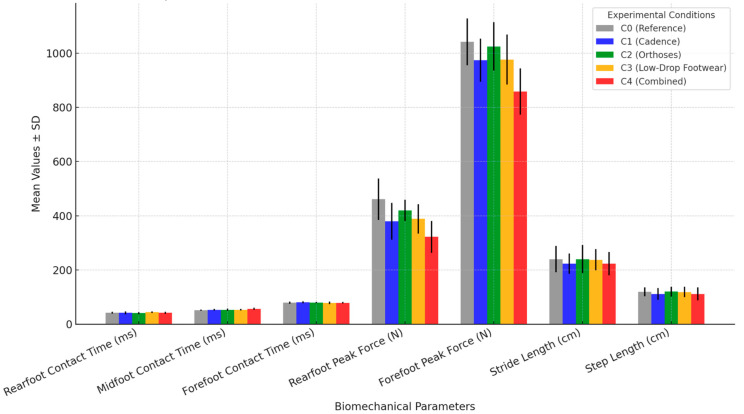
Distribution of biomechanical parameters across different running conditions.

In terms of peak rearfoot force, all conditions led to a significant reduction, with C4 producing the most pronounced decrease (−139.09 N, Cohen’s d = 1.02, *p* < 0.001). Similarly, for peak forefoot force, C4 showed the greatest reduction (−183.18 N, Cohen’s d = 1.21, *p* < 0.001), indicating a substantial effect. Finally, regarding stride and step length, both C1 and C4 significantly reduced stride length by −17 cm and −16.6 cm, respectively (Cohen’s d = 0.56 and 0.54, *p* < 0.05), suggesting an adaptation toward a more compact running pattern (Figure 3).

## 4. Discussion

The aim of our study was to assess the individual and combined effects of different interventions on key spatiotemporal parameters of strength and force distribution. Our findings contribute to the growing body of literature on running biomechanics and provide practical insights for optimizing running mechanics to reduce injury risk and enhance performance.

Regarding the drop of the shoes, these parameters, in the scientific literature, are influenced by the various experimental conditions. A shoe with a 10 mm drop (C0) favors a rearfoot attack, increasing rearfoot contact time and peak rearfoot force. However, the impact on stride and step length may vary between individuals [36]. Our results align with previous studies, which demonstrated that a higher rearfoot-to-toe drop promotes a rearfoot strike pattern, increasing loading on the knee and hip joints [25,37].

A 10% increase in cadence (C1) generally reduces ground contact time, decreases peak force, and can slightly shorten stride length, improving efficiency and reducing the risk of injury [24]. Increasing cadence by 5–10% significantly reduces impact forces and loading rates, particularly at the knee joint [38].

Inversion foot orthoses (C2) alter the distribution of plantar pressures, influencing peak forces and contact times in different areas of the foot, with varying effects depending on the correction made [39]. Orthotics play a key role in redistributing plantar loads and reducing stress on specific structures, such as the medial tibia and Achilles tendon [40].

The use of shoes with a drop reduced to 5 mm (C3) encourages a midfoot or forefoot attack, reducing rearfoot contact time and peak rearfoot force, while possibly increasing forefoot contact time [36]. Minimalist shoes with low rearfoot-to-toe drops promote a forefoot strike pattern, reducing impact forces at the rearfoot but increasing loading on the forefoot [41]. Finally, a combination of several of these conditions (C4) with a +10% cadence, inversion soles, and shoes with a 5 mm drop can encourage a midfoot attack, reduce ground impact, and optimize force distribution, positively influencing stride and step length [36,39]. A multifactorial approach is essential to address the complex biomechanical demands of running, supporting the need for integrated interventions [42].

This study demonstrates that increasing cadence (C1) significantly reduces stride length and step length, decreases peak force in the rearfoot and forefoot, and increases contact time in the midfoot and forefoot. These findings align with research indicating that increased cadence reduces impact forces and stride length, enhancing running economy and reducing injury risk [43]. Our findings have significant implications for clinical and training practices. For example, increasing cadence (C1) could be particularly beneficial for runners with high injury rates, such as those with patellofemoral pain syndrome (PFPS) or iliotibial band syndrome (ITBS), as it reduces knee-joint loading and impact forces [38].

Alain Lavigne Inversion Foot Orthoses (C2) did not significantly affect stride or step length but reduced peak rearfoot force, with no significant forefoot changes, and increased midfoot and forefoot contact time. This suggests that orthotics redistribute plantar loads, primarily affecting the rearfoot without altering stride length [44]. Alain Lavigne Inversion Foot Orthoses (C2) could be particularly useful for runners with excessive pronation or flat feet as they help redistribute plantar pressures and reduce stress on the medial tibia and Achilles tendon.

Using shoes with a reduced drop (5 mm) (C3) led to moderate reductions in stride and step length, decreased rearfoot and forefoot peak forces, increased midfoot contact time, and reduced forefoot contact time. These effects are consistent with studies showing that a lower shoe drop encourages a midfoot strike, altering impact forces and running mechanics [45].

The combination of these interventions (C4) resulted in pronounced reductions in stride and step length, significant decreases in rearfoot and forefoot peak forces, and increased midfoot contact time, with no changes in forefoot contact. This cumulative effect underscores the benefits of integrated approaches, as highlighted in literature advocating for combined strategies to maximize biomechanical benefits [46].

Overall, the findings corroborate prior research showing that biomechanical adjustments, such as increasing cadence and using reduced-drop shoes, enhance running economy and decrease lower-joint impact forces, reducing injury risk [36,43]. Orthotics contribute by redistributing forces without affecting stride length, supporting existing research on their role in managing plantar load distribution [47]. Specific interventions, like cadence increases (C1) and reduced-drop shoes (C3), could be recommended as non-invasive strategies to reduce mechanical stress on lower joints, benefiting individuals with conditions such as plantar fasciitis, medial tibial stress syndrome, or Achilles tendinopathy [10,48]. ALIFOrthoses (C2) may assist in redistributing plantar loads, aiding in rehabilitation after injuries such as stress fractures or foot deformities (for example, flat or hollow foot) [49].

Combining these interventions (C4) could optimize multiple biomechanical parameters in personalized rehabilitation protocols, particularly for complex cases involving multiple injury risk factors or performance limitations. Furthermore, these strategies can be integrated into motor reprogramming programs to teach optimized running techniques, offering proactive solutions for injury prevention and recurrence [50].

This study has several limitations that should be acknowledged. First, the sample size of 23 participants, although sufficient for detecting significant differences, may limit the generalizability of the findings. A larger sample size would enhance the statistical power and allow for subgroup analyses based on factors such as running experience or injury history.

Second, the short duration of the intervention (two minutes per condition) may not fully capture the long-term effects of these biomechanical adjustments. Future studies should consider longer intervention periods to assess the sustainability of these changes.

Third, the controlled laboratory setting, while ideal for standardizing conditions, may limit the ecological validity of the findings. Running on a treadmill differs from outdoor running in terms of surface variability, environmental factors, and psychological responses. Future research should validate these findings in real-world settings using wearable sensors or outdoor running protocols.

This study paves the way for further research into the multifactorial optimization of running biomechanics. Future studies could explore the longitudinal effects of these combined interventions on sports performance and injury prevention. For example, a six-month follow-up study could assess whether the observed biomechanical changes lead to a reduction in injury rates or improvements in running economy.

Additionally, it would be relevant to evaluate these adjustments in specific populations, such as runners with musculoskeletal injuries, by integrating clinical and functional parameters to assess the benefits on performance and overall health. For instance, runners with a history of stress fractures or Achilles tendinopathy could be targeted to evaluate the effectiveness of reduced-drop shoes and orthotics in reducing recurrence rates.

Another promising direction is the integration of wearable technologies, such as on-board sensors or smart insoles, to provide real-time feedback on running mechanics. These devices could be used to validate treadmill findings in outdoor running conditions and to personalize biomechanical adjustments based on individual needs. For example, a runner with excessive pronation could receive real-time feedback on foot strike patterns and pressure distribution, enabling immediate corrections during training sessions.

Our results indicate that cadence adjustments (+10%) and low-drop footwear (5 mm) can significantly reduce rearfoot impact forces, making them practical, non-invasive interventions for injury prevention. Orthotic modifications, while less influential on stride parameters, effectively redistribute plantar pressures, making them useful for runners with existing biomechanical imbalances:

- Cadence adjustment (+10%) may be particularly beneficial for runners at high risk of overuse injuries (e.g., patellofemoral pain syndrome (PFPS) or iliotibial band syndrome (ITBS)), as it helps reduce knee-joint loading [38].
- Inversion foot orthoses (C2) could be useful for runners with excessive pronation or flat feet, as they aid in redistributing plantar pressures and reducing stress on the medial tibia and Achilles tendon.
- Low-drop footwear (C3) may help runners transition to a midfoot strike, potentially benefiting those with high-impact running mechanics but requiring careful adaptation to avoid excessive forefoot loading.
- Combined interventions (C4) could be recommended for runners needing comprehensive biomechanical optimization, particularly in rehabilitation settings or performance training programs.

## 5. Conclusions

This study shows that modifying cadence (+10%) and wearing reduced-drop footwear (5 mm) significantly influences running mechanics, particularly by reducing impact forces and altering foot contact durations. ALIF Orthoses (C2) had a limited effect on stride length but significantly redistributed plantar loads, suggesting its role in load management rather than stride modification.

The combined intervention (C4) produced the most pronounced cumulative effects, particularly in reducing impact forces and modifying foot contact durations, reinforcing the benefits of a multifactorial approach in optimizing running biomechanics.

Future research should assess the long-term impact of cadence adjustments and footwear modifications on injury rates, particularly in runners with a history of overuse injuries. Additionally, wearable technology could be leveraged to monitor real-time biomechanical adaptations in natural running environments

Combining these interventions (C4) offers a multi-faceted strategy to enhance running efficiency, optimize biomechanics, and reduce injury risk.

This study highlights the potential of combined biomechanical modifications in enhancing running mechanics and reducing injury risks. By integrating cadence, footwear, and orthotic adjustments, runners and clinicians can implement evidence-based strategies for performance optimization and injury prevention.

## Figures and Tables

**Table 1:** Each condition’s key features.

Condition	Footwear	Drop	Cadence	Orthoses
C0 (Reference)	Control shoes	10 mm	No adjustment (0%)	None
C1 (Cadence only)	Control shoes	10 mm	+10%	None
C2 (Plantar orthoses)	Control shoes	10 mm	No adjustment (0%)	ALIFOrthoses
C3 (Asics Noosa)	Asics NOOSA	5 mm	No adjustment (0%)	None
C4 (Cross-interventions)	Asics NOOSA	5 mm	+10%	ALIFOrthoses

**Table 2:** Comparison of spatiotemporal parameters and force distribution across different running conditions.

Parameter	C0 (Mean ± SD)	C1 (Mean ± SD)	C2 (Mean ± SD)	C3 (Mean ± SD)	C4 (Mean ± SD)	Δ (vs. C0)	*p*-Value	Effect Size (Cohen’s d)
Rearfoot contact time (ms)	42.22 ± 3.45	42.00 ± 5.34	41.00 ± 4.23	44.11 ± 2.87	41.83 ± 3.76	−1.22 (C2), +1.89 (C3)	<0.001 *	0.45 (C2), 0.61 (C3)
Midfoot contact time (ms)	51.45 ± 2.3	53.28 ± 2.67	53.27 ± 4.22	53.43 ± 3.29	56.52 ± 3.98	+1.83 (C1), +5.07 (C4)	<0.001 *	0.48 (C1), 0.89 (C4)
Forefoot contact time (ms)	79.4 ± 4.32	80.45 ± 3.4	80.09 ± 2.39	78.5 ± 4.2	79.02 ± 3.1	+1.01 (C1), −0.94 (C3)	<0.001 *	0.35 (C1), 0.38 (C3)
Rearfoot peak force (N)	461.08 ± 76.5	379.72 ± 67.8	419.77 ± 38.8	388.95 ± 54.4	321.99 ± 59.2	−81.36 (C1), −139.09 (C4)	<0.001 *	0.55 (C1), 1.02 (C4)
Forefoot peak force (N)	1041.7 ± 86.4	973.65 ± 79.2	1024.48 ± 89.3	976.63 ± 92.2	858.54 ± 85.2	−68.07 (C1), −183.18 (C4)	<0.001 *	0.47 (C1), 1.21 (C4)
Stride length (cm)	240.17 ± 48.2	223.17 ± 37.3	240.12 ± 52.2	237.69 ± 39.1	223.57 ± 42.9	−17.00 (C1), −16.60 (C4)	<0.001 *	0.56 (C1), 0.54 (C4)
Step length (cm)	119.75 ± 16.3	111.41 ± 21.7	119.99 ± 18.4	118.50 ± 19.3	111.67 ± 23.9	−8.34 (C1), −8.08 (C4)	<0.001 *	0.52 (C1), 0.50 (C4)

* Significant; ms milliseconds; cm centimeter; N newton; SD Standard deviation.

## Data Availability

The data are unavailable due to privacy or ethical restrictions; a statement is still required.
